# Using flow technologies to direct the synthesis and assembly of materials in solution

**DOI:** 10.1186/s13065-016-0229-1

**Published:** 2017-01-05

**Authors:** K. Robertson

**Affiliations:** Department of Chemistry, University of Bath, Bath, BA2 7AY UK

**Keywords:** Microfluidics, Continuous crystallisation, Mesofluidics, Microreactor

## Abstract

In the pursuit of materials with structure-related function, directing the assembly of materials is paramount. The resultant structure can be controlled by ordering of reactants, spatial confinement and control over the reaction/crystallisation times and stoichiometries. These conditions can be administered through the use of flow technologies as evidenced by the growing widespread application of microfluidics for the production of nanomaterials; the function of which is often dictated or circumscribed by size. In this review a range of flow technologies is explored for use in the control of self-assembled systems: including techniques for reagent ordering, mixing control and high-throughput optimisation. The examples given encompass organic, inorganic and biological systems and focus on control of shape, function, composition and size.

**Graphical abstract:**
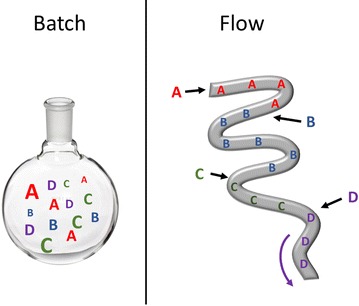
.

.

## Background

The use of flow technologies for chemical applications has become a fast growing area with a wide range of reaction types identified as having benefited from flow processing [1]. Flow environments are used to achieve conditions not accessible in batch such as: very fast or very slow mixing of reagents; ordering of reagents; physical confinement for control of geometry/habit; highly repeatable reaction/crystallisation conditions; isolation of reactants/products and use of very small volumes of reagents (pl–μl). These conditions are interlinked and are inherent to the nature of flow environments; for example, the ability to crystallise reproducibly material of a specific size or polymorph is reliant on the control of mixing conditions and temperature. The manner in which this can be achieved is dependent on the scale of the reactor; microreactors have excellent mixing properties usually induced by bends in the channels creating Dean vortices ensuring steady-state operation, mesoreactors require additional mixing elements such as segmentation for Taylor flow or static mixers e.g. Kenics type. As such the different scale of reactor is dictated by the application. Mesoreactors are more applicable for scale-up production of exquisite particles and crystallisation of particles incompatible with microreactors. Microreactors have the advantage of using very small volumes making them ideal for high-throughput applications for synthesis or assembly of expensive or precious materials at low volume. The control over fluid dynamics is outstanding in microreactors enabling the construction of very precisely controlled architectures such as spherical particles or foams. This review will highlight the different areas in which flow technologies have enabled the synthesis and directed-assembly of materials in both meso and microreactors.

## Introduction to meso and microfluidic reactors

**Fig. 1:**
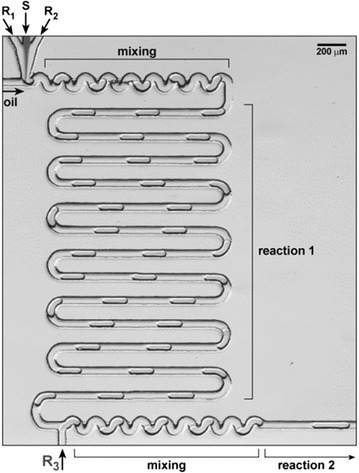
Example of a flow chemistry chip with liquid segmented flow, *R*_*1–3*_ reagents, *S* separating fluid (prevents premature reaction between R_1_ and R_3_. Reprinted with permission from Ref. [2]. Copyright 2004 The Royal Society of Chemistry

There is a wide range of different flow reactors, some with very specific designs for their applications. In general microreactors are based around the standard flow chemistry chip (Fig. 1) where the small channel size (width ~10–500 µm) means a simple t-junction can lead to excellent mixing while bends in the channel create Dean vortices which generate further mixing along the reactor length. These can be used in monophasic flow (single net stream) or segmented flow arrangements. Segmented flow is where there are two or more immiscible phases (liquid/liquid, gas/liquid etc.) producing discrete droplets (slugs) of solution. This can be used to impart a variety of properties e.g. mixing (via non-slip boundary between immiscible phases), isolation of droplets or templating.

**Fig. 2:**
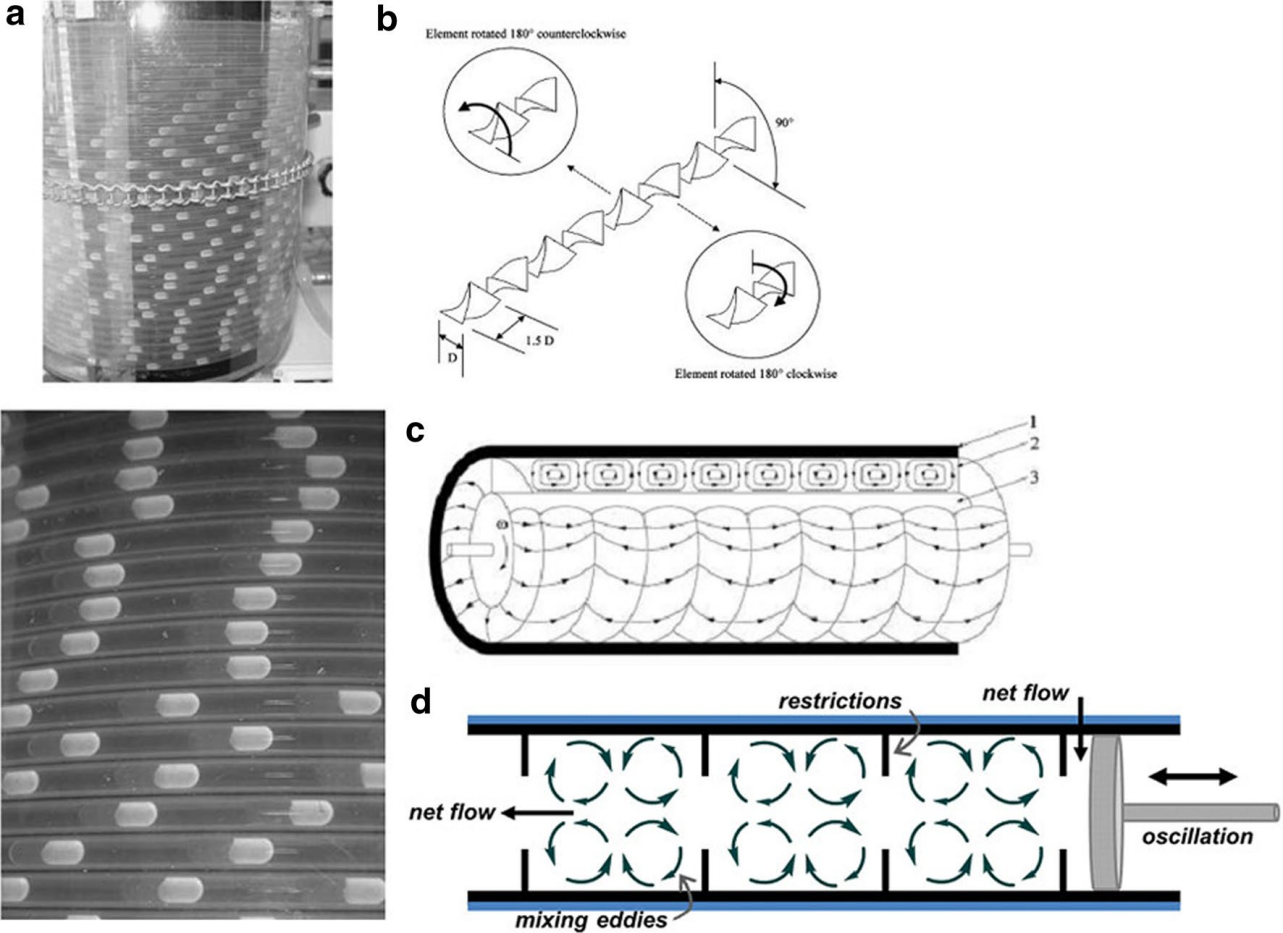
**a** Open tubular meso-reactor with liquid segmented flow. Reprinted with permission from Ref. [3]. Copyright 2003 Elsevier B.V. **b** Kenics-type insert for mixing in tubular reactors. Reprinted with permission from Ref. [5]. Copyright 2010 American Chemical Society, **c** Couette–Taylor crystalliser highlighting vortexes. Reprinted with permission from Ref. [6]. Copyright 2012 American Chemical Society, **d** COBC showing eddie formation with oscillation. Reprinted with permission from Ref. [7]. Copyright 2015 American Chemical Society

Mesoreactors have a wider variety of designs for similar applications. This is because the excellent mixing properties of microreactors are diminished upon increasing the size of the channels and so a variety of engineering solutions have been devised to counterbalance this. Open tubular meso-reactors (up to 4.6 mm channel width, Fig. 2a) are essentially scaled-up versions of microreactors. Whilst monophasic examples exist [3], typically open tubular mesoreactors employ segmented flow as otherwise at this scale back-mixing becomes a significant problem [4]. By inserting static mixing elements into the tubing both mixing and back-mixing issues can be mitigated (Fig. 2b). Couette-Taylor devices comprise two interposed cylinders with solution filling the annular void, the internal barrel rotates creating vortices in the solution establishing the turbulent flow pattern shown in Fig. 2c. Oscillatory baffled reactors (e.g. continuous oscillatory baffled crystallisers, COBC) have a net flow of solution which is passed through a series of constrictions (baffles). A piston then oscillates the flow so that around each baffle eddies are created leading to turbulent mixing of solution (Fig. 2d).

These variations in design are employed to ensure that all of the solution passing through the reactor experiences a homogeneous environment (mixing and temperature conditions) for any given point of the reactor. Evaluation on the efficacy of each reactor towards homogeneity can be performed by injecting tracer solutions [8] or by evaluating the homogeneity of the resultant product. In flow crystallisation experiments this is confirmed by the particle size distribution (PSD), the range of particles sizes obtained from each experimental run of the reactor. A narrow PSD implies a high level of homogeneity within the reactor and is typically the goal for flow crystallisation; this has the benefit that, if the size of product can be controlled, an experiment can potentially be designed to produce a targeted particle size.

## Control of self-assembled shape

**Fig. 3:**
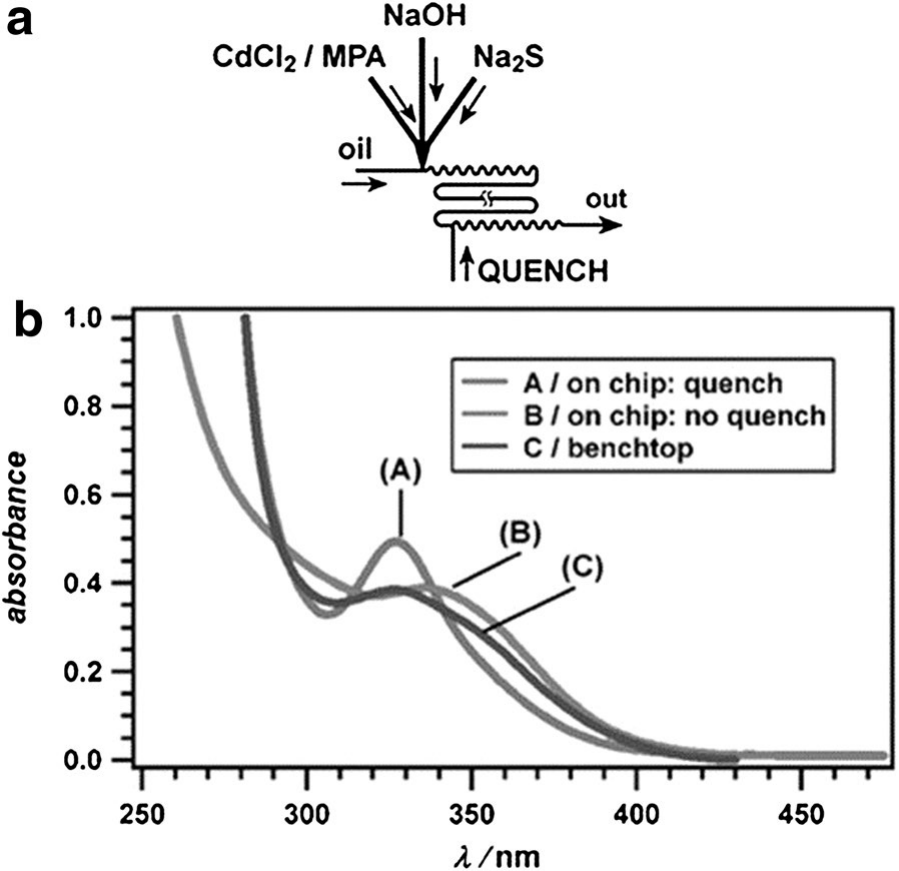
**a** Schematic representation of microfluidic for quenched CdS nanoparticle production, **b** UV–Vis spectra showing sharper absorbance for quenched nanoparticles than non-quenched. Reprinted with permission from Ref. [2]. Copyright 2004 The Royal Society of Chemistry

Microfluidics for crystallisation has largely been used in the production of nanoparticles and nanowires. It is not hard to see why, as the typical size of microreactors does not allow for the growth of much larger particles and the control over temperature and mixing can exact a size control unachievable in batch processes. The size of nanoparticles is very important, as often the intensity of response of the nanoparticle to stimuli is directly correlated to its size [9]. Using on-line UV–vis or photoluminescence measurements, the Maeda [10] and Bawendi [11] groups were able to identify the conditions under which a narrow PSD CdSe nanoparticles (widely used for sensors) [12] can be obtained using the exquisite temperature control of a monophasic microreactor. Liquid-segmented flow has successfully been used to produce nanoparticles with a narrow PSD [13]. Of particular interest is the work by Ismagilov et al. where a multi-step process was used to produce nanoparticles of CdS in liquid segmented flow with a second inlet which contained quench solution (1-mercaptopropionic acid) [2]. This ensured not only that the desired particle size was obtained within the reactor but that prolonged exposure of the nanoparticles to growth solution during downstream processing (filtering, washing and centrifuging) did not affect the PSD (Fig. 3). By changing the quench solution to Na_2_Se very high quality core–shell nanoparticles of CdS/CdSe could be obtained, utilising the ability to tandem reactions in flowing environments.

**Fig. 4:**
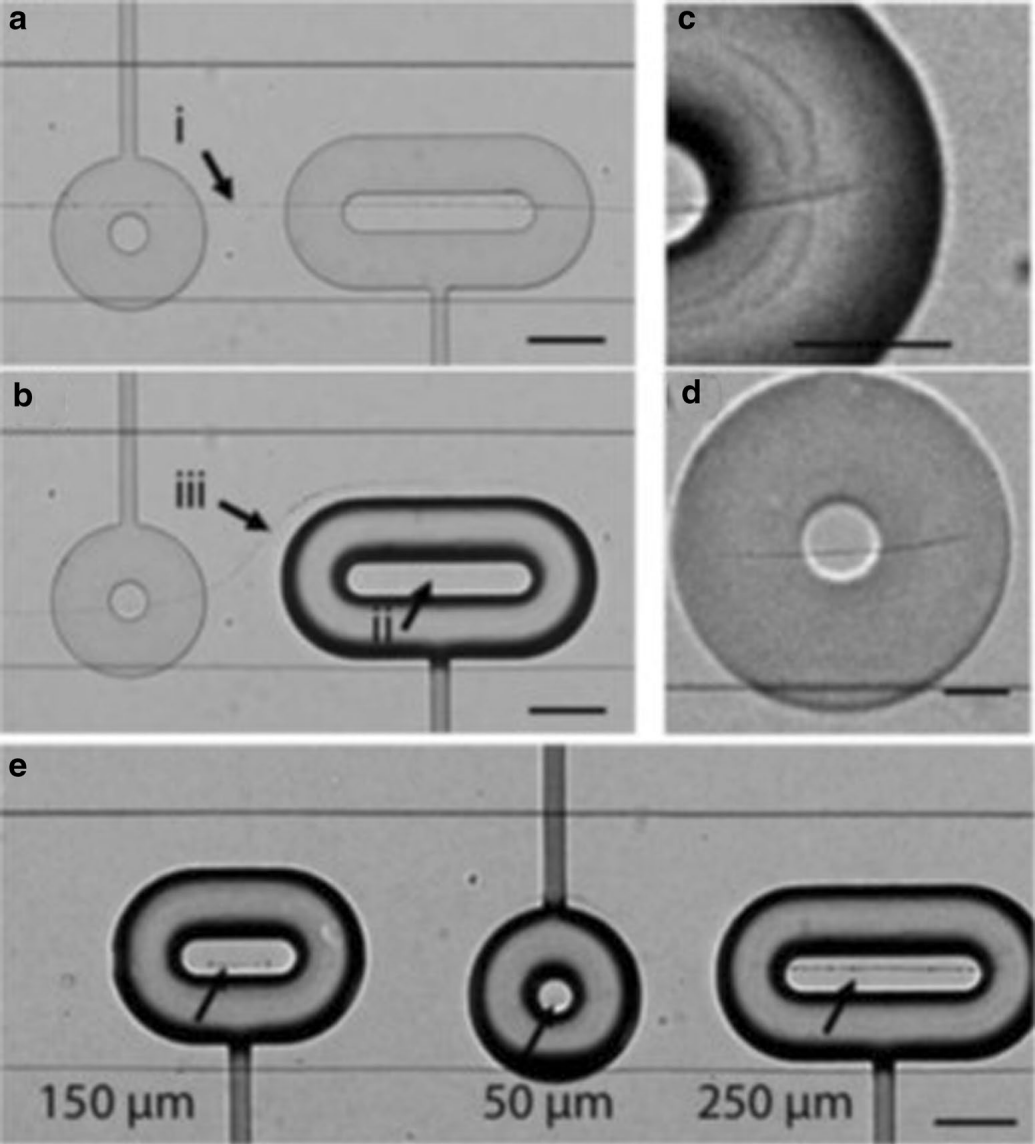
**a** Formation of nanowires at solution boundary, **b** entrapment of nanowires through activation of donut-shaped clamp, **c** close-up of trapped nanowire under donut clamp, **d** nanowires stay in position after careful deactivation of clamp under non-flowing conditions, **e** highlighting different sizes of nanowire bundles achievable by use of various clamp shapes. *Scale bars* **a**, **b**, **e** 100 μm and **c**, **d** 50 μm. Reprinted with permission from Ref. [15]. Copyright 2011 The Royal Society of Chemistry

**Fig. 5:**
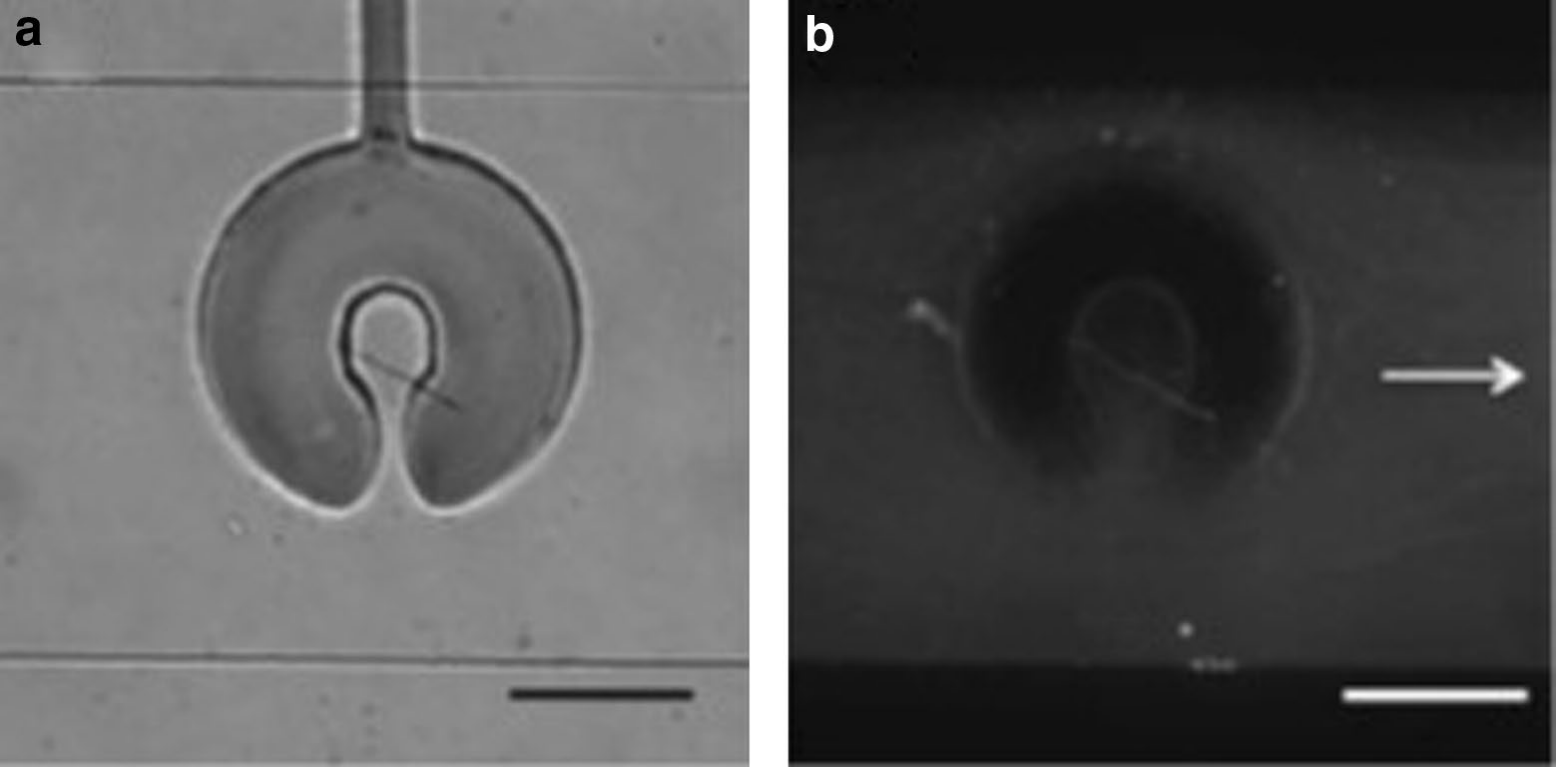
**a** Trapped TTF-Au nanowire in clamp with small opening, **b** fluorescence imaging showing successful post-synthetic functionalisation. *Scale bars* 100 μm. Reprinted with permission from Ref. [15]. Copyright 2011 The Royal Society of Chemistry

Flow focusing of a single reagent stream by anti-solvent streams or two reagent streams by carrier fluid can lead to the precipitation of nanoparticles/wires in a confined area away from the walls of the crystalliser, preventing fouling and maximizing recovery. Génot and co-workers evaluated the effect of different magnitudes of flow, focussing on the size of the resultant nanoparticles [14]. By flowing anti-solvent either side of a solution of rubrene at various flow rates this method proved able to manipulate either a broad or narrow precipitation zone and thus tailor the size of rubrene nanoparticles produced. Dittrich et al. produced bundles of Cu^II^Asp nanowires by co-flowing solutions of Cu(NO_3_)_2_ and l-aspartic acid (Asp), subsequent use of pneumatic clamps trapped the nanowire bundles and allowed them to be cut into desired lengths (Fig. 4) [15]. The microreactor used comprised a baseplate with solution channels, a polydimethylsiloxane (PDMS) membrane and a top plate with ‘donut’ shaped clamps which, once gas filled, pressed down onto the solution plate trapping both solution and nanowire bundle. By altering the design of the clamp to include an inlet, post-synthetic functionalisation was carried out on trapped bundles of in situ produced TTF-Au (TTF = tetrathiafulvalene) nanowires. Once trapped the nanowires were exposed to fluorescent carboxylate nanoparticles which bound to the TTF-Au nanowires, illustrated by fluorescent microscopy (Fig. 5).

**Fig. 6:**
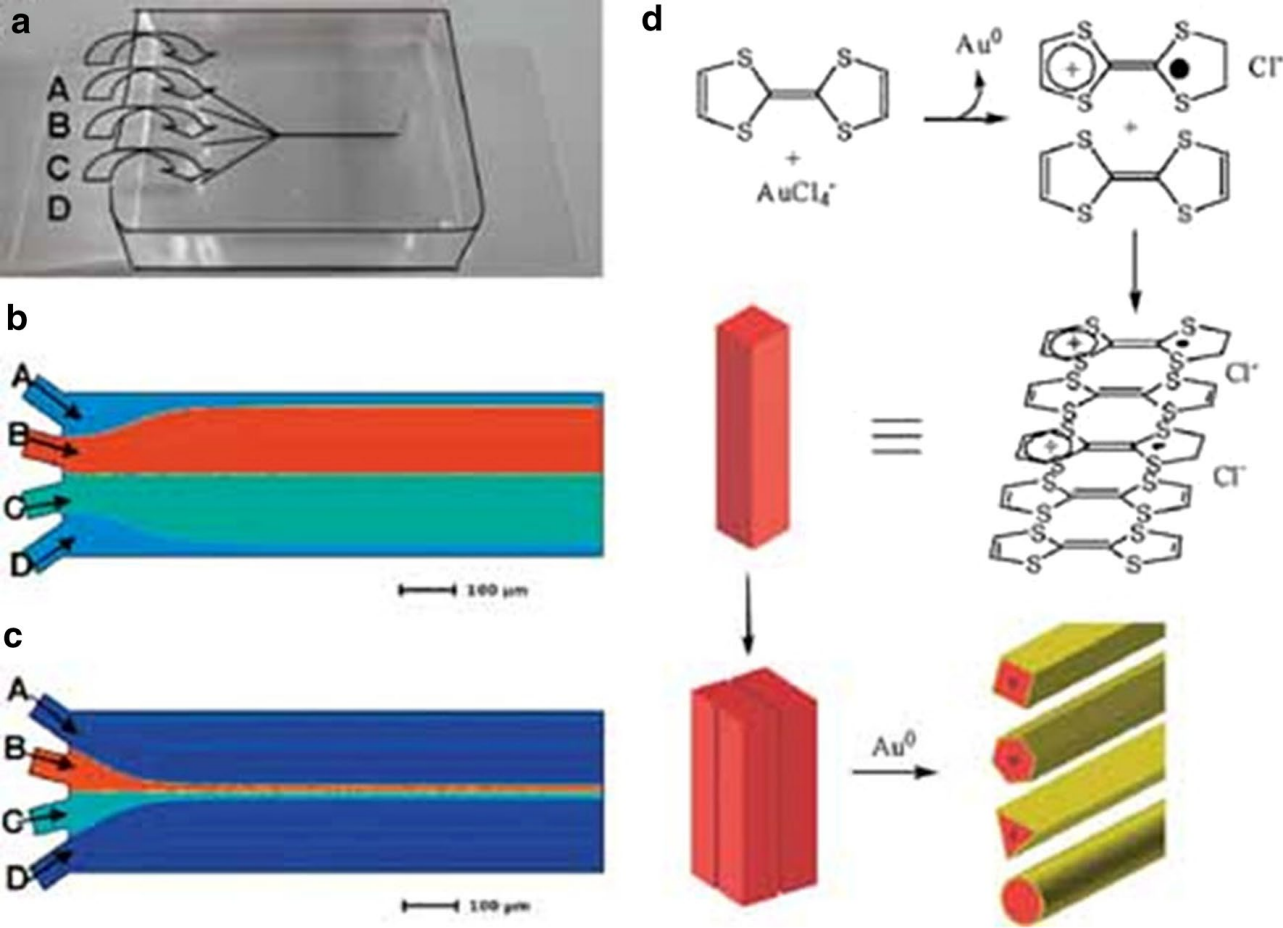
**a** Reactor used for nanowire synthesis showing inlets, **b** simulated flow of sheath (*A* and *C*) and reagent flows at a ratio of 0.1, **c** ratio 10, **d** schematic representation of hollow nanowire assembly. Reprinted with permission from Ref. [16]. Copyright 2010 WILEY–VCH Verlag GmbH & Co

Puigmartı´-Luis and co-workers used flow focussing techniques to produce TTF-Au nanowires of specified shapes [16]. By changing the ratio of inert sheath/side flow rates and reagent stream flow rates long nanowires, nanorods or block crystals were observed. Even more interestingly, by agglomeration of single nanowires, hollow nanowires of cylindrical, cuboidal, hexagonal and triangular geometries were observed (Fig. 6). These hollow nanowires represent a step forward in the production of targeted architectures with potential uses in encapsulation and sensors.

**Fig. 7:**
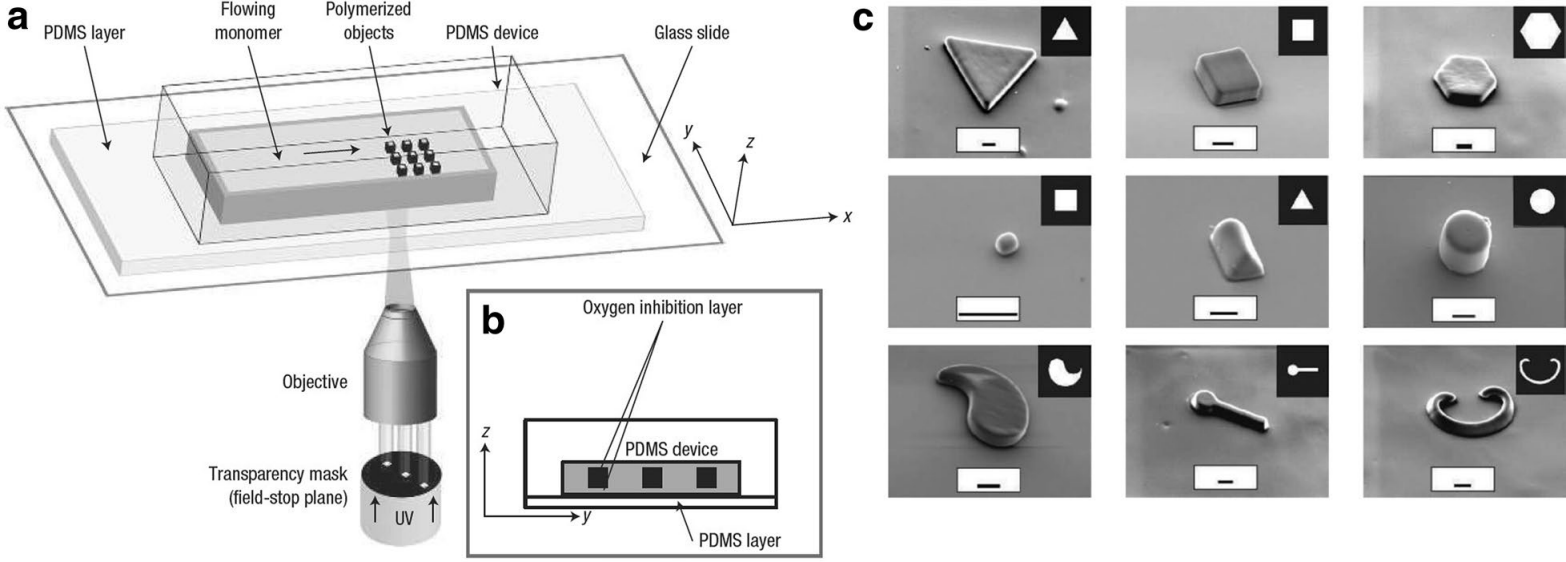
**a** Schematic view of the stopped flow lithography (SFL) microfluidic device developed by Doyle et al. **b** Polymerisation is prevented at the interface of the reactor walls due to oxygen inhibition. This prevents any fouling and enables the particles to be washed downstream with resumed flow, **c** range of PEG-DA particles synthesised (insets show transparency masks used for each shape, *scale bars* are 10 μm), the height is determined by the microfluidic channel. All images are copyright Nature Publishing group and reprinted with permission from Ref. [17]

**Fig. 8:**
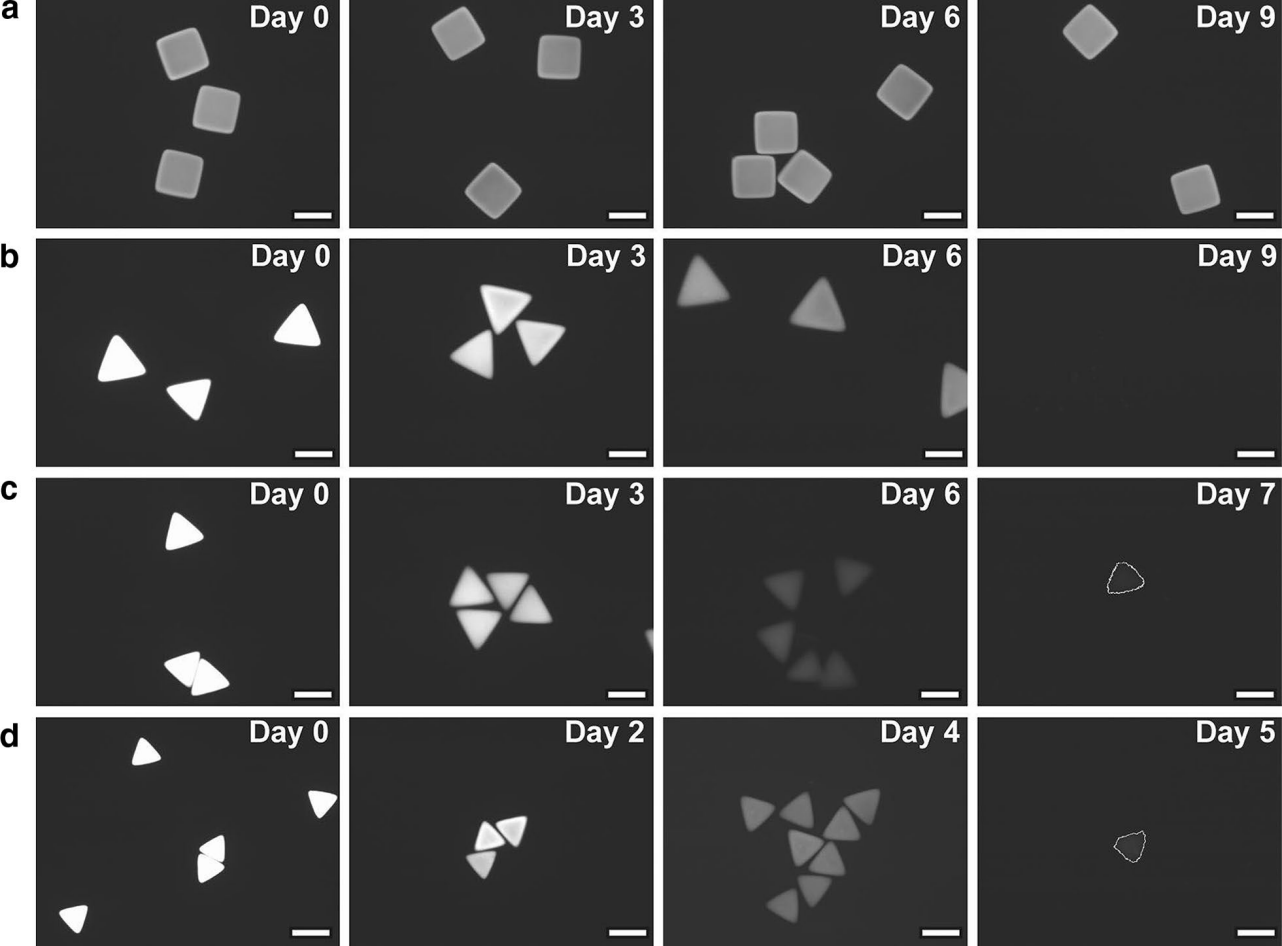
Fluorescence imaging of microgels showing degradation of gel over time; **a** non-degradable PEG-DA control, **b–d**. 30, 20, 10 wt% PEG-PLA. *Scale bars* are 50 μm. Images are reprinted with permission from Ref. [18] Copyright 2009 American Chemical Society

In order to generate micro-materials of reproducible and confined geometry, Doyle et al. have developed the technique of stopped-flow lithography (SFL) [17]. In a stopped-flow microfluidic device, successive aliquots of oligomer solution (poly(ethylenegylcol)-diacrylate, PEG-DA) were exposed to micro-focussed UV radiation when the flow is stationary, polymerising the solution (Fig. 7a). The geometric shape of the resultant polymeric material is dictated by the transparency mask through which the UV-beam is passed (Fig. 7c). Once the solution has been polymerised the flow is resumed, washing the shaped polymer materials downstream and delivering fresh solution ready for polymerisation. A wide range of microgels of co-block polymers poly(ethylene)glycol (PEG) and polylactic acid (PLA) were formed using this technique, offering homogeneous and controllable degradation rates (Fig. 8) [18]. Such gels have direct applications in progressive drug delivery.

**Fig. 9:**
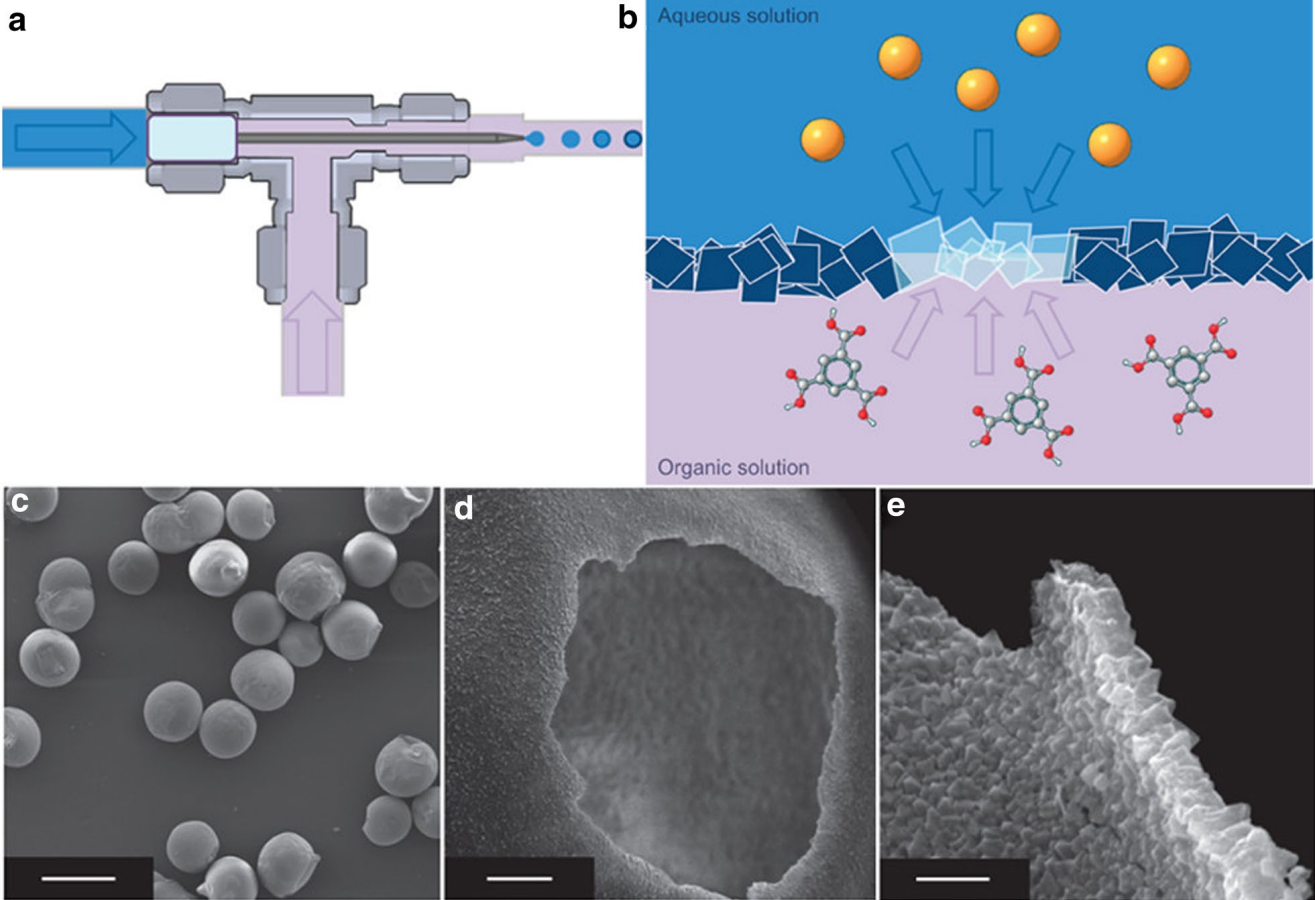
**a** Schematic of microdroplet generation, **b** schematic of coordination polymer self-assembly at solution interface, **c**–**e**. SEM images of hollow MOF spheres (*scale bars* 500, 25 and 5 µm, respectively) nanocrystalline agglomeration visible in **e**. Reprinted with permission from Ref. [19]. Copyright 2011 Macmillan Publishers Limited

Due to the geometry of microreactors it is an obvious progression to see applications in the production of microspheres through liquid segmentation. Microspheres can be used for encapsulation of materials e.g. for drug delivery or heterogenising of homogeneous catalysts, production of low density material and biomimetics. By using the oil solubility of benzenetricarboxylate (BTC) and aqueous solubility of cupric acetate, Vos and co-workers formed hollow spheres of the coordination polymer [Cu_3_(BTC)_2_] [19]. The sphere is comprised of agglomerated nanocrystals to produce a porous membrane which can be used for encapsulation and catalysis (Fig. 9).

**Fig. 10:**
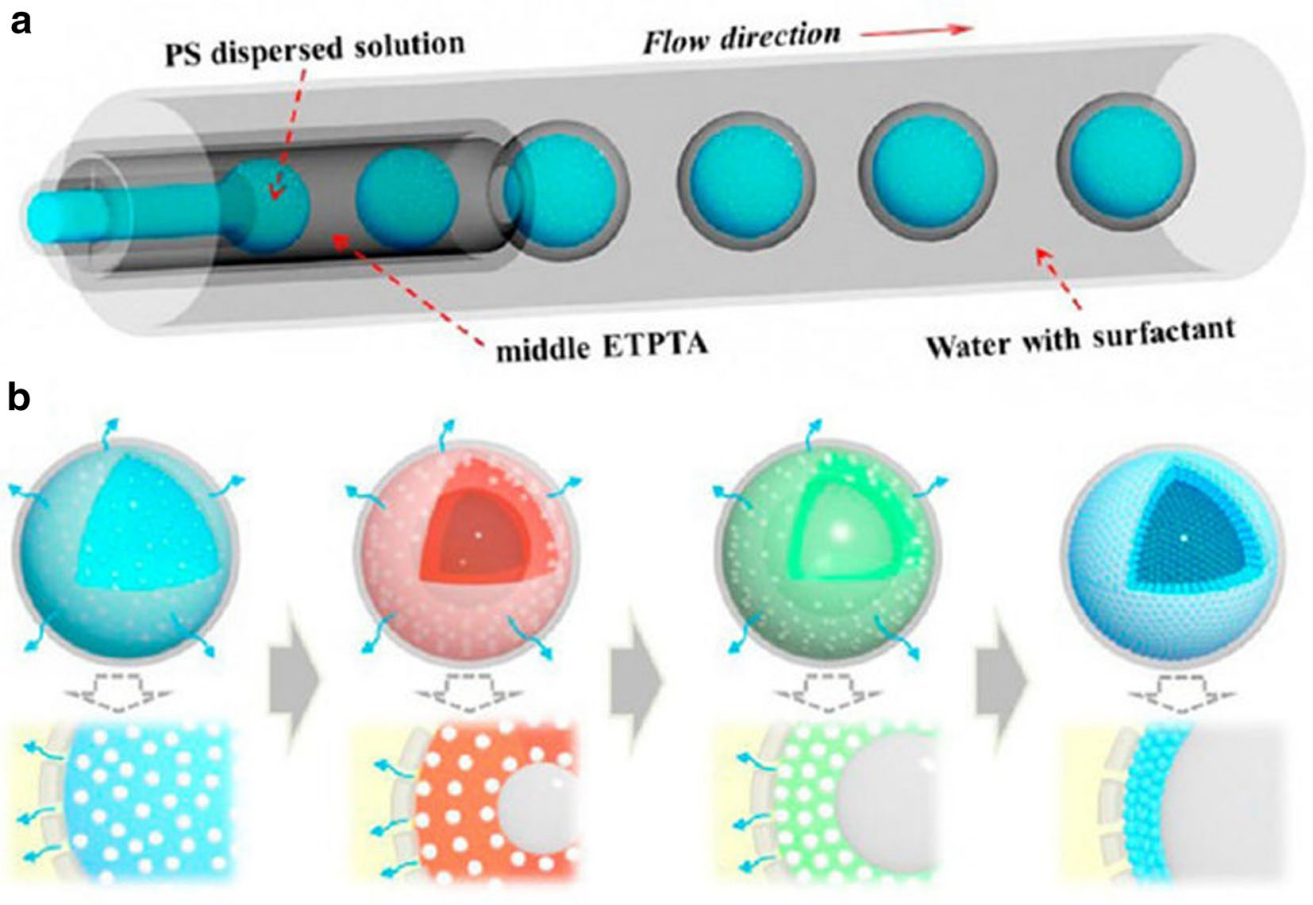
Schematic representation of (**a)** multi-layered microdroplet generation, **b** assembly of hollow PhC spheres through concentration, extraction and drying processes. Reprinted with permission from Ref. [20]. Copyright 2015 American Chemical Society

Photonic crystals (PhCs) are promising candidates as barcode-particles for multiplexed high-throughput bioassays but have a high density and so poor suspension properties. By creating hollow spheres of PhCs by flow methods, Gu et al. were able to impart a low density on these functional materials [20]. In this approach, photocurable ETPTA resin (with 1% photoinitiator) is first segmented by an aqueous suspension of polystyrene (PS) nanoparticles with surfactant which is subsequently encapsulated by a further aqueous surfactant solution producing multi-layer spheres. These are then solidified by curing via UV irradiation downstream to produce microspheres of PhCs (Fig. 10). The microspheres display a variable density (depending on whether gas or liquid filled) enabling suspension of material without detriment to the desirable surface properties of PhCs.

**Fig. 11:**
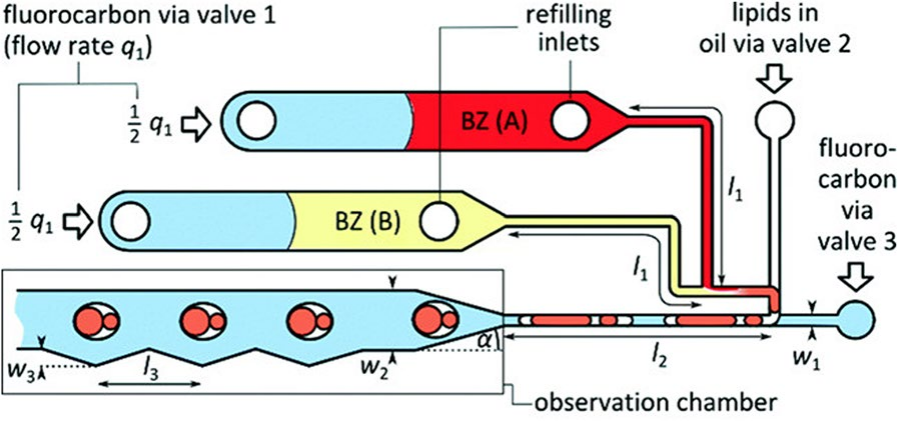
Schematic representation of microfluidic chip and asymmetric droplet generation. The notches in the walls of the observation chamber trap droplets for monitoring purposes. Reprinted with permission from Ref. [23]. Copyright 2016 The Royal Society of Chemistry

**Fig. 12:**
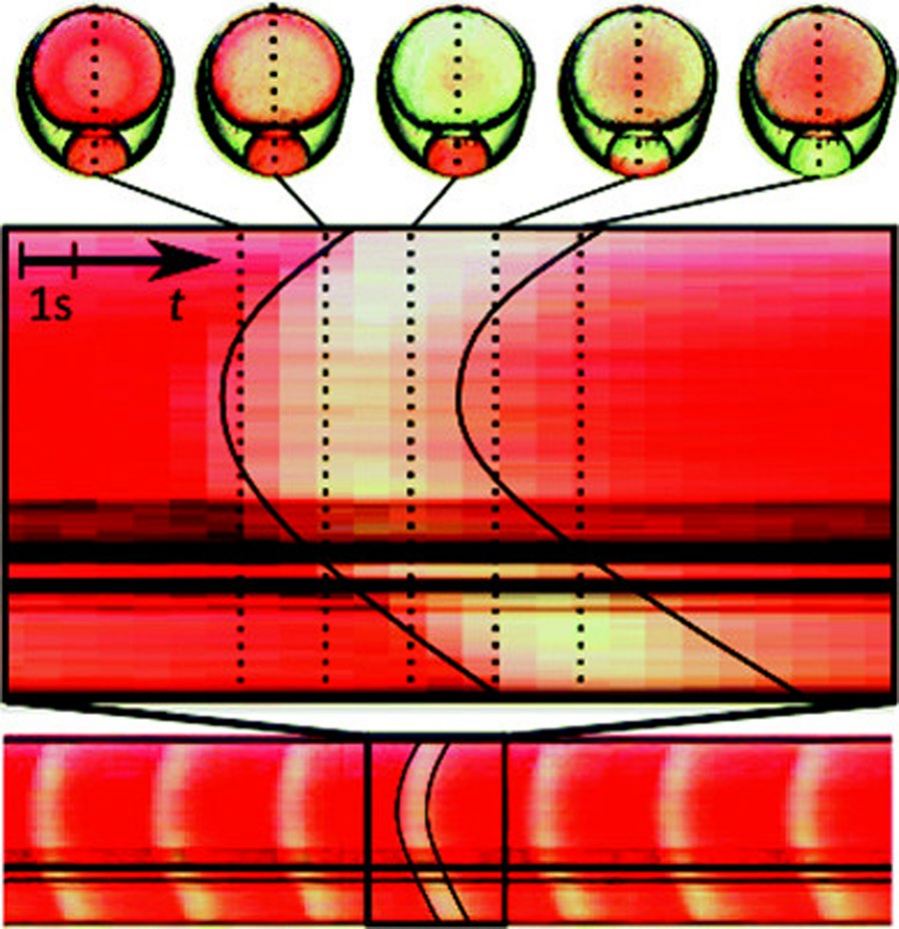
Time resolved chemical communication between BZ droplets observed by colour change. The parabolic shape illustrates the chemical wave projecting outward from the centre (most concentrated region) of the droplet. Reprinted with permission from Ref. [23]. Copyright 2016 The Royal Society of Chemistry

The investigation of transport of materials through cell-membranes is a very important challenge for biochemistry with implications for e.g. drug delivery [21]. It can, however, be difficult to synthesise a representative system for investigation. By generating liquid-segmented flow with aqueous primary amines and carboxylated perfluorocarbons, Easley et al. created biocompatible emulsions which were evaluated using homogeneous protein assays, droplet polymerase chain reaction (PCR) and droplet recombinase polymerase amplification (RPA) [22]. Garstecki and co-workers investigated cell transport by generating multi-compartment droplets with bilipid membranes encapsulating Belousov–Zhabotinsky (BZ) solution [23]. The droplets were formed by encapsulation of two asymmetric droplets of aqueous BZ solution in a mixture of lipids and subsequent liquid segmentation by fluorocarbon oil creating a transparent and stable barrier for isolated membrane transport studies (Fig. 11). BZ solution comprises a reversible catalytic reaction which displays a change in colour due to the adoption of different oxidation states of the catalyst; transport between the pseudo-cells is therefore illustrated by a change in colour of the droplet (Fig. 12). The effect of relative size of droplets and ratio of BZ pre-cursor solutions can be investigated quickly and simply by altering flow rates.

**Fig. 13:**
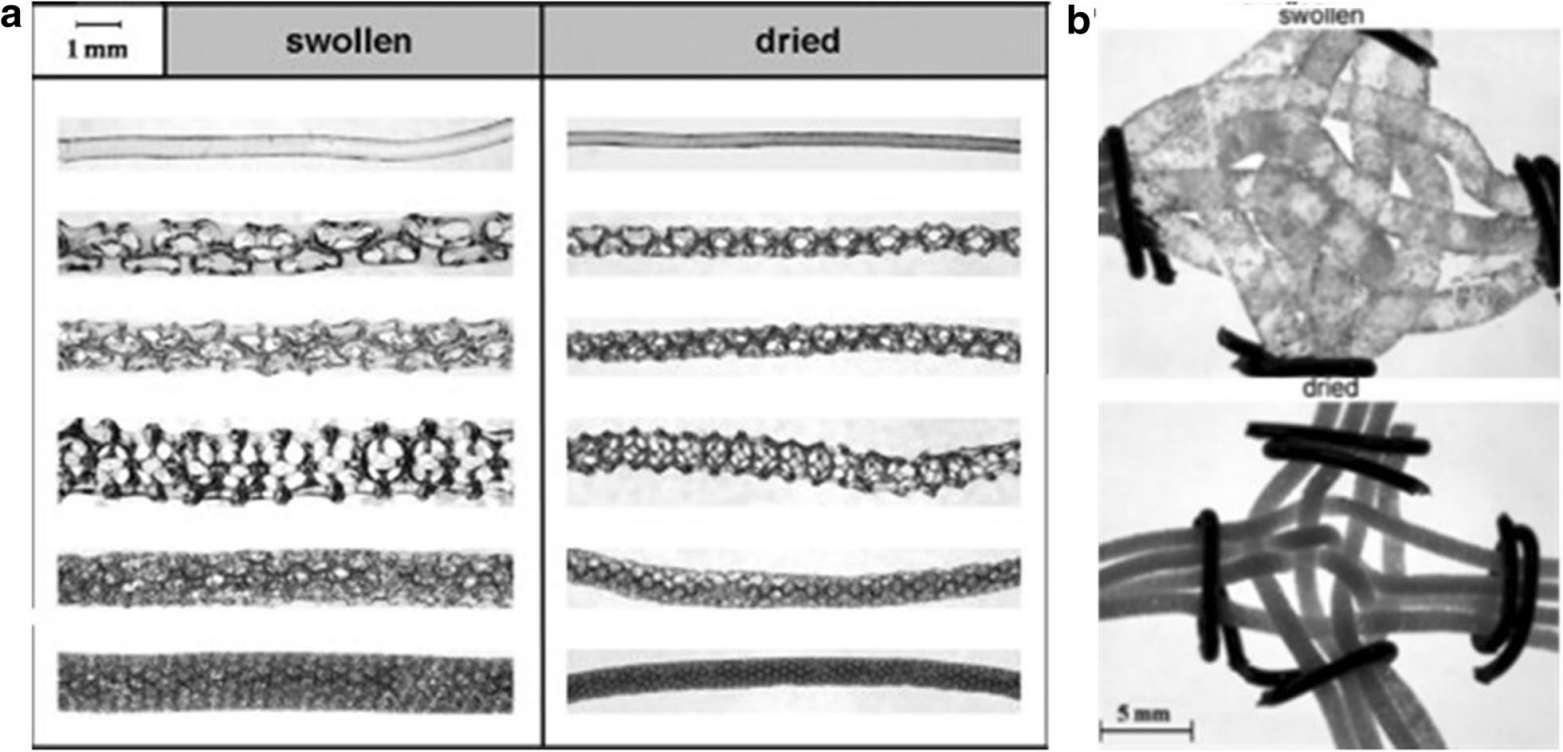
**a** Swollen and dried threads of hydrogel with varying pore size and connectivity, **b** vertices of interwoven foam threads. Reprinted with permission from Ref. [26]. Copyright 2009 Elsevier B.V

**Fig. 14:**
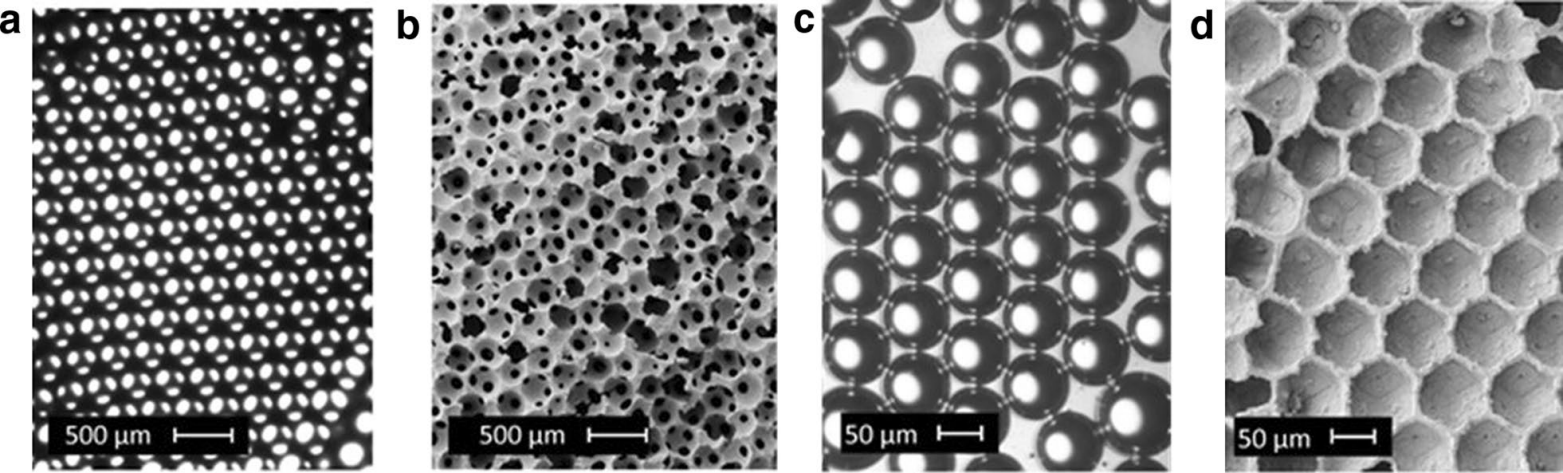
Liquid (**a**, **c**) and solidified (**b**, **d**) foams of polystyrene generated through flow techniques. Reprinted with permission from Ref. [28]. Copyright 2015 WILEY–VCH Verlag GmbH & Co

In addition to the production of discrete droplets, microreactors are ideal for the generation of an array of bubbles, i.e. foams. The precise nature of the foam can be tuned by the relative flow rates of the gas and liquid, and the subsequent confinement area [24]. The generation of foams can facilitate investigation of crystal packing [25] and the production of functional materials with regular and designed porous architectures. By combining two flows of polymerisation solutions at a cross-piece with a net flow of air, Drenckhan et al. produced stable foams of hydrogel which can be dried and re-swollen repeatedly [26]. The connecting vertices of these foams show crosslinking of the polymers which adds to the stability of the foam (Fig. 13). In later work more rigid foams were generated using polyurethane [27] or polystyrene [28], 3D dry foams were obtained with a connectivity and pattern directly related to the bubble density (determined by the liquid: gas ratio and net flow rate) and drying period (Fig. 14).

## Production of functional substrates

The activity of surface active sensing techniques such as localised surface plasmon resonance (LSPR) and surface enhanced Raman spectroscopy (SERS) is highly dependent on the size and homogeneity of the nanoparticles which make up the substrate [29, 30]. The production of substrates with highly homogeneous nanoparticles of desirable particle size and shape is therefore of the utmost importance for progressing these techniques.

**Fig. 15:**
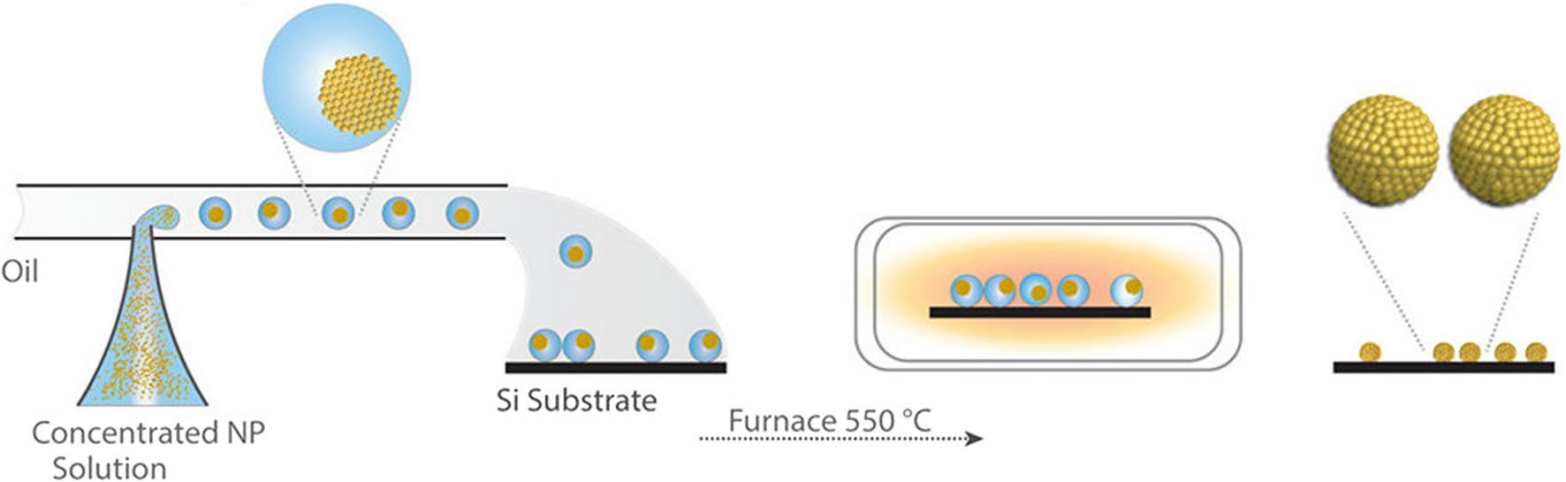
Schematic illustration of the assembly of solid Au microspheres from concentrated nanoparticle (NP) solution through microdroplet generation and subsequent calcination. Reprinted with permission from Ref. [31]. Copyright 2015 The Royal Society of Chemistry

A combination of nanoparticle and microsphere production was used to generate solid Au microspheres by Edel et al. [31]. A concentrated non-agglomerated feed solution of nanoparticles was created by centrifugation of a nanoparticle solution [32] which was segmented by oil to produce droplets, the size of which dictated the size of microspheres (Fig. 15). As the microspheres are an agglomeration of nanoparticles, the surface area is very high, a property that makes the Au microspheres particularly effective as substrate material for SERS.

## Directing solutions for printing and high-throughput applications

**Fig. 16:**
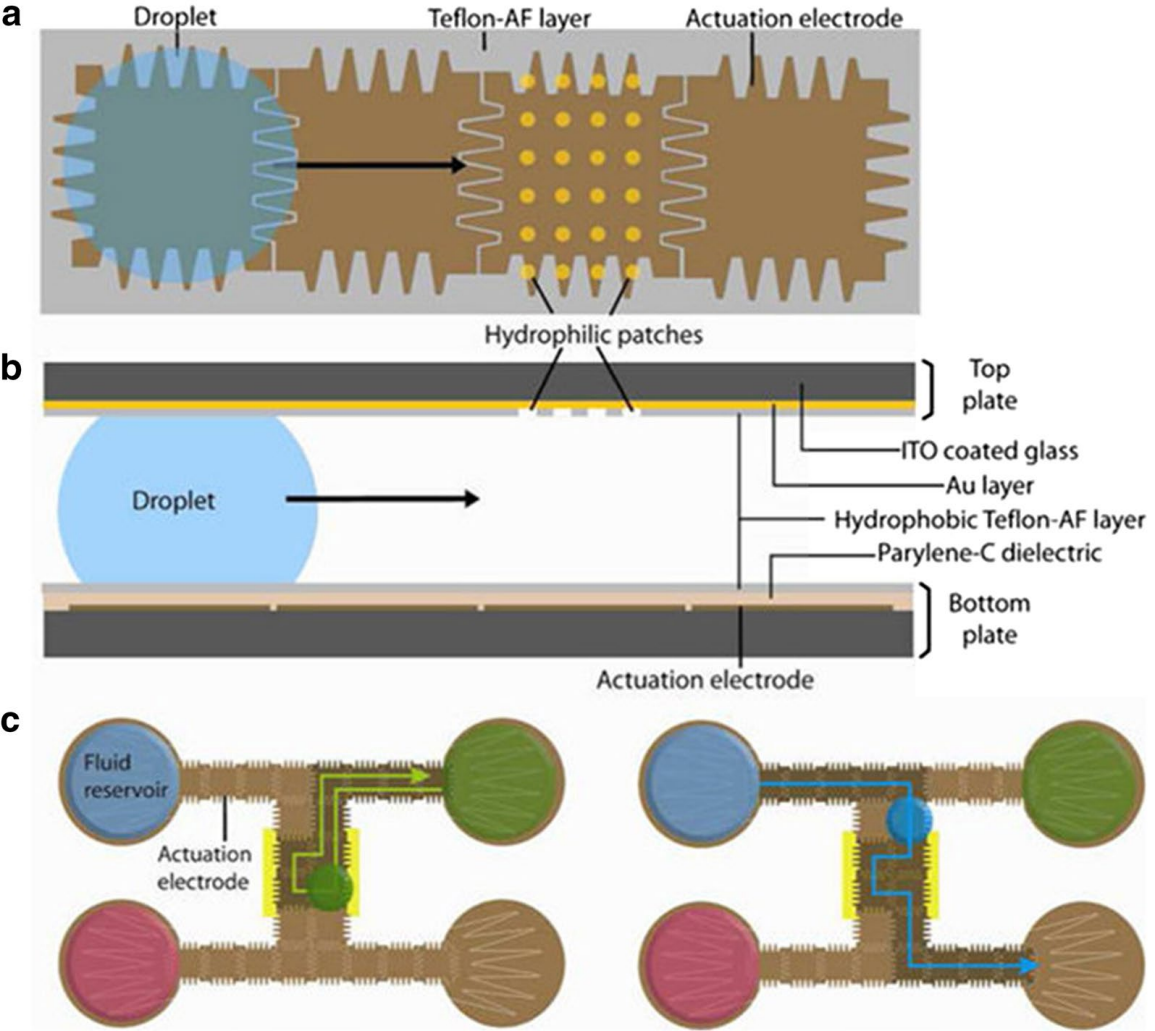
*Top* (**a**) and side (**b**) views of microfluidic EWOD chip highlighting hydrophilic patches designed for controlling deposition of materials on desired regions only, **c** illustration of sequential deposition and washing through control of droplet path by electrowetting. Reprinted with permission from Ref. [19]. Copyright 2013 American Chemical Society

So far we have only discussed linear flow through tubing or single channels, while with microfluidics it is possible to direct the flow of solutions in 2D space. By using the preferential wetting properties of different solvents, droplets of solution in segmented flow can be ‘docked’ allowing isolation. Valve-based microfluidic devices can isolate aliquots of solution of very carefully defined geometries. Mechanical manipulation of a microfluidic chip can allow the combination of microwells of solution in a simple but effective manner. Due to the small size of droplets in a microfluidic chip, electrical impulses can be used to guide the droplets over a 2D grid in a very methodical manner. Digital microfluidics is a fast developing area in which aqueous solutions on a hydrophobic surface can be manipulated by the application of an electrical current. Electrowetting on dielectrics (EWOD) uses the attraction of aqueous solutions to an electrical charge to very precisely direct the flow. Ameloot and co-workers used this technique to create thin films of the metal–organic framework (MOF) HKUST-1 in a specified location by manipulating solutions of ligand, metal salt and a wash solution alternately over a targeted area within a mircoreactor, ensuring that the whole area for deposition was covered by the roaming droplet (Fig. 16) [19].

**Fig. 17:**
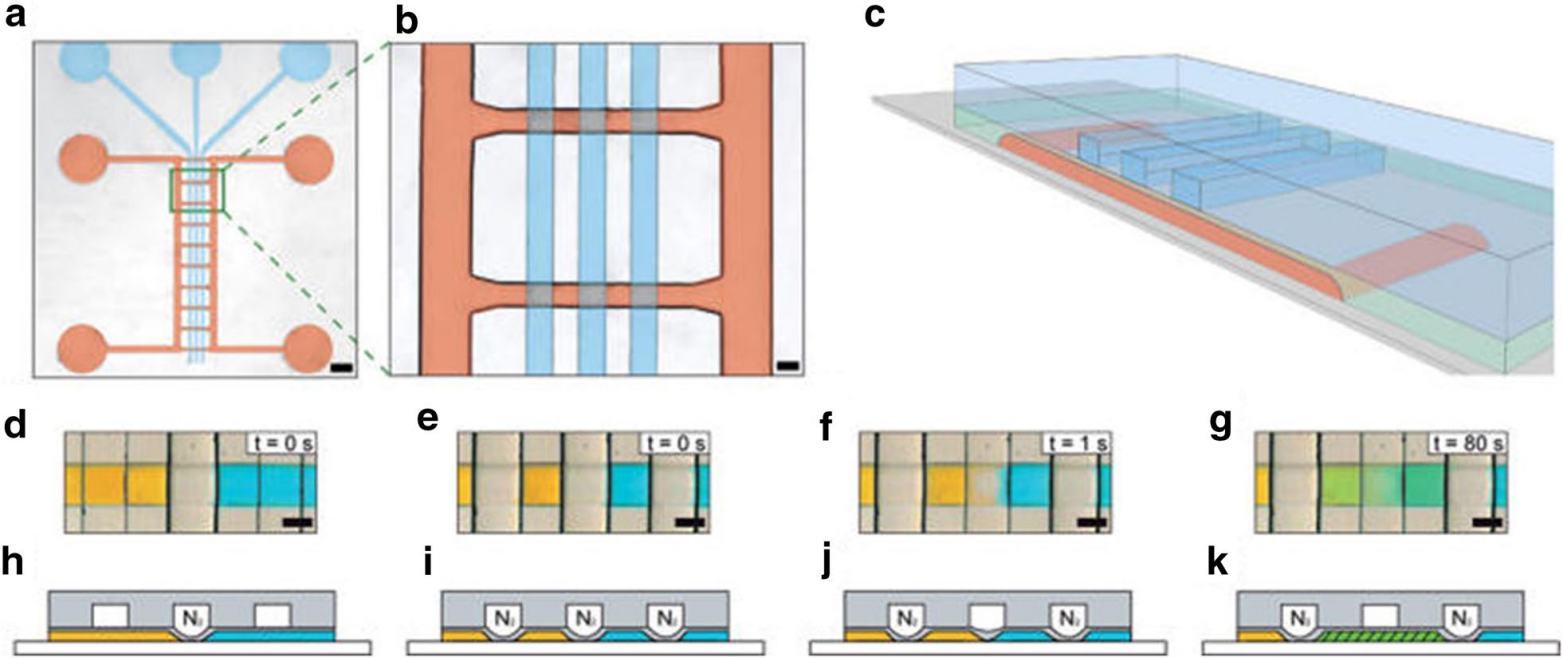
**a** Micrograph of the microfluidic plate with pneumatically operated valves to isolate solutions, **b** close-up of two ‘rungs’ with overlapping gas channels (*blue*) for solution isolation, **c** schematic representation highlighting the vertical positioning of the gas channels, **d–g** micrographs of two coloured solutions separated, isolated and subsequently mixed by activation and deactivation of the pneumatic valves, **h**–**k**. Reprinted with permission from Ref. [33]. Copyright 2012 American Chemical Society

Following on from successful work exploiting pneumatic clamps for nanowire synthesis, Dittrich et al. used a microfluidic plate with a ladder-like appearance, in which each strut is a reagent flow that can combine on the ‘rungs’ [33]. With gas filled polydimethylsiloxane (PDMS) membranes, isolated cells can be created, capturing the two reagent streams and enabling diffusion of specific volumes of solution. Nanowires of AgTCNQ (TCNQ = tetracyanoquinodimethane) were synthesised by first reacting solutions of silver salt with a reducing agent; with the pneumatic clamps activated, the reagent streams were washed and replaced with TCNQ solution which was then introduced to the Ag^o^ nanowires by deactivation of one of the clamps, ensuring slow diffusion and promoting the transformation to AgTCNQ (Fig. 17).

**Fig. 18:**
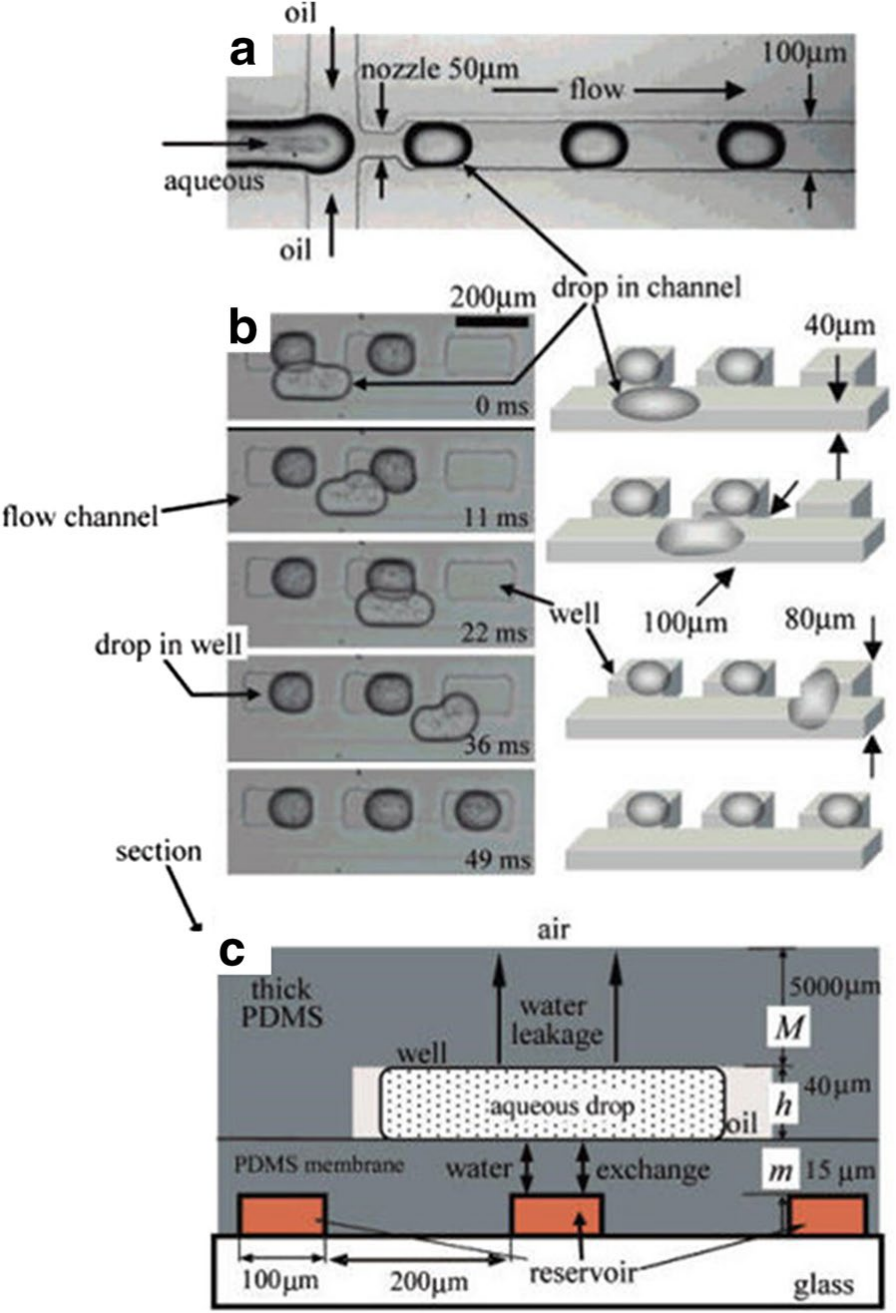
**a** Image of the flow focussing droplet generator used in the Phase Chip, **b** series of images and illustrations showing docking of droplets. As a droplet passes an empty well the change in surface tension resulted by an increase in height within the well relative to the channel creates the docking effect (N.B. the channel and well sizes differ in the chip used in this example to chips with reservoir), **c** side-view illustration of phase chip with reservoir showing permeation through the PDMS membrane between docked droplet and reservoir below. Reprinted with permission from Ref. [34]. Copyright 2007 American Chemical Society

**Fig. 19:**
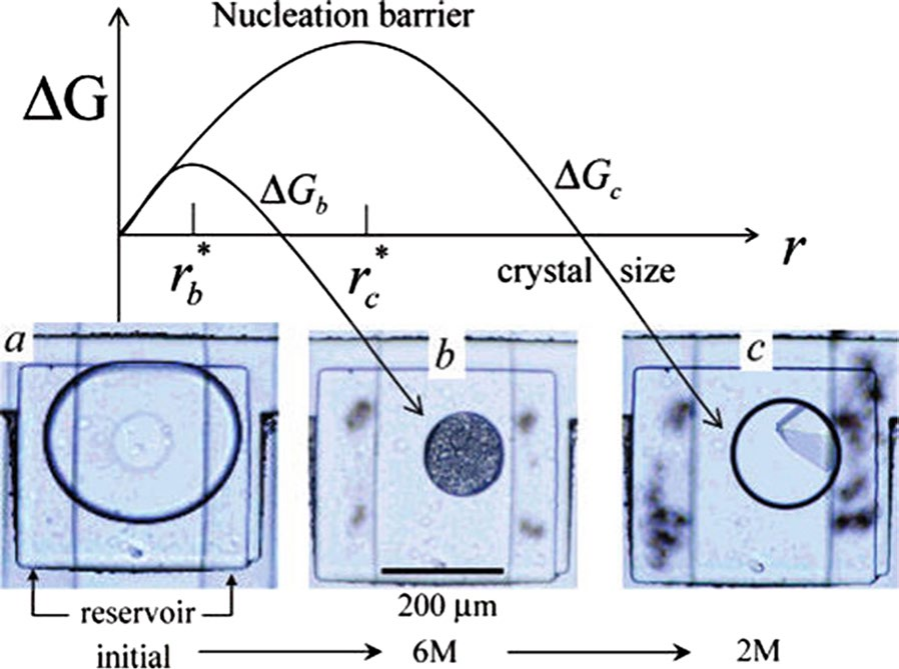
Schematic illustration of the free-energy barrier to nucleation as a function of crystal size, **a** micrograph image of a docked droplet, **b** the reservoir is filled with 6 M NaCl solution causing water to transfer from the droplet into the reservoir causing precipitation of poor quality nanocrystallites, **c** the reservoir is filled with 2 M NaCl solution resulting in some re-dissolution of protein and subsequent nucleation of a single crystal. Reprinted with permission from Ref. [34]. Copyright 2007 American Chemical Society

The isolation of droplets can also be achieved by ‘docking’. This method uses the surface tension properties of droplets to manoeuvre them out of the course of the net flow. One example of the application of this method is the ‘phase-chip’ developed by the Fraden group, in which wells were created off the main channel flow. In these wells, the height was slightly greater than in the channel; droplets would therefore fall into these wells due to the alleviation of surface tension, while subsequent droplets pass by until reaching an empty well (Fig. 18) [34]. The base of the flow side of the phase chip is a PDMS membrane allowing transport of water between the docked droplets and the aqueous stream on the opposing side of the membrane. By changing the salt concentration of the aqueous flow the concentration of protein solution in the droplets could be tuned, enabling the solubility limit of the proteins to be found and thus controllably produce single crystals (Fig. 19). Protein crystallisation is well-known for the challenges involved, and for this reason much of the research effort in microfluidic crystallisation has been directed at this area [35–40].

**Fig. 20:**
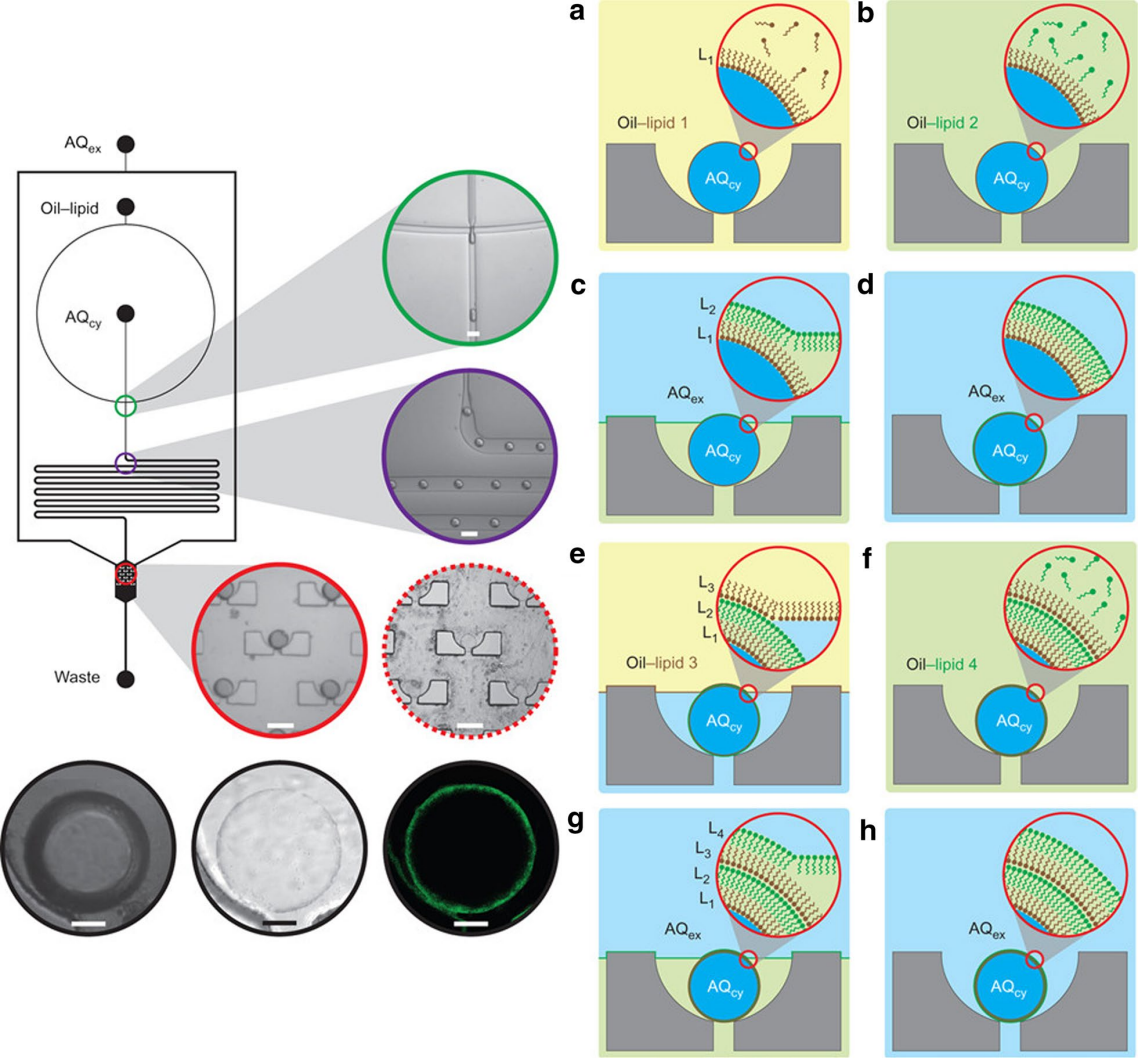
Schematic of the chip used to create double bi-layered vesicles, inserts are micrographs highlighting the various stages of vesicle production; droplet generation, transport and docking. After lipid deposition the contrast between vesicle and surrounding fluid is weakened; *bottom* images show docked droplet before and after lipid deposition and with fluorescent labelled lipid layer. Schematic illustration of (**a**) lipid deposition on docked droplet of cytoplasmic fluid stabilising the droplet, **b** exchange of lipid solution to oil–lipid 2, **c–d** as oil–lipid 2 is exchanged with extracellular aqueous solution the first lipid bilayer is formed, **e**–**h** second lipid bi-layer formed through systematic replacement of surrounding fluid. Reprinted with permission from Ref. [36, 41]. Copyright 2013 Macmillan Publishers Limited

In similar vein, Paegel et al. created a series of ‘cups’ at the base of a microfluidic reactor in which droplets of aqueous solution could be docked whilst oil was able to flow past the droplets without displacing them [41]. This device was used to generate multi-layered cell-like spherical membranes by passing successive oil-solubilised lipids and finally extracellular proteins over the cytoplasmic droplets (Fig. 20). These synthetic cells were then able to assemble a pore-forming protein complex and display basic cell-like metabolism functions after DNA insertion. The array of synthetic cells generated in this way can then be used for studies into the transport of material across a cell membrane and consequent metabolic functions, in a controlled and repeatable manner.

**Fig. 21:**
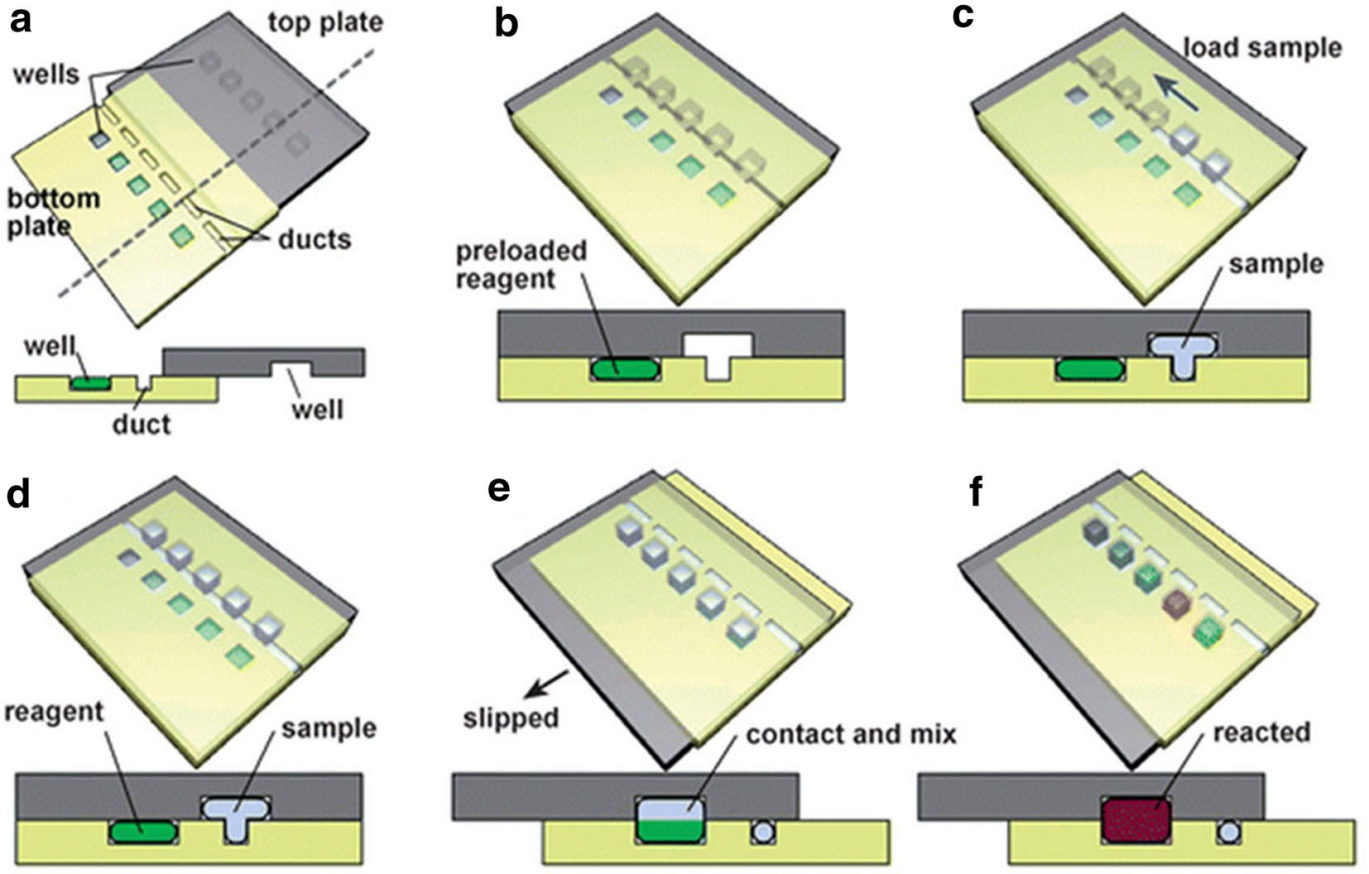
Schematic illustrastion of the slip-chip showing **a** loading of reagents, **b** open channel created for automatic sample loading, **c–e** transfer of sample to reagent wells (volume of sample is controlled by sample well dimensions) by ‘slipping’ the top half of the chip into scendary position, **f** reaction of sample with reagents. Reprinted with permission from Ref. [36]. Copyright 2009 The Royal Society of Chemistry

Flow technologies are particularly well suited to high-throughput optimisation applications; using small volumes of materials to investigate a wide range of parameters such as co-former [42], concentration [34] and reaction time [38]. Ismagilov and co-workers have developed an array of microfluidic devices for high-throughput optimisation of crystallisation conditions [43, 44]. The slip-chip consists of microwells which can be pre-loaded with various solutions and a top-plate with channels which can be filled with the constant parameter solution. The two plates, once loaded, can be combined by slipping them together so that specified volumes of the top solution diffuse into the isolated microwells (Fig. 21) [36].

Alternative designs to the slip-chip allow automatic loading of precursors and enable either comparison of reagent ratios by combining differing well sizes [39] or investigation of crystallisation kinetics by incorporating channels of differing lengths between the two microwells of solution [40]. A similar approach to the optimisation of crystallisation kinetics of proteins was devised by Quake et al., in which a splitter directs the flow of reagent into successive channels with differing lengths [38].

## Timescales unattainable in batch conditions

**Fig. 22:**
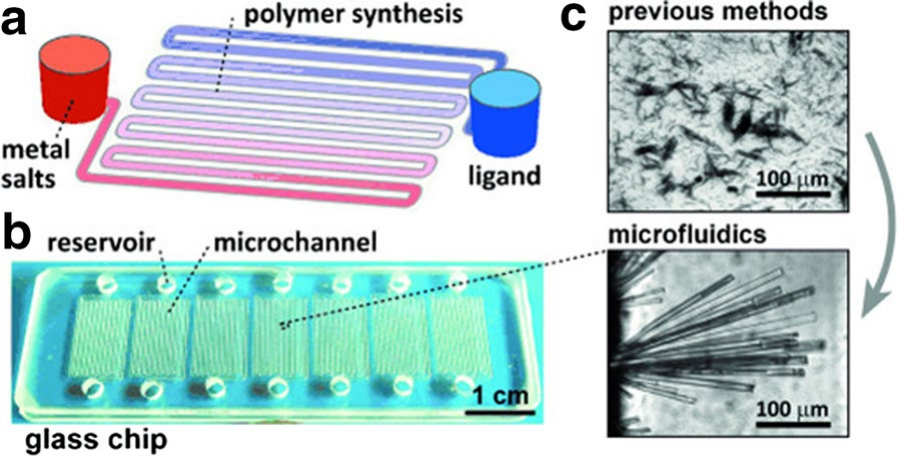
**a** Illustration of microreactor used for the slow diffusion of reagents, channels are filled with oil through which each reagent slowly diffuses, **b** image of reactor plate with seven parallel reactors, **c** microscopy images of crystals of silver phenylacetylide formed by common laboratory methods (*top*) and using the microfluidic diffusion chip (*bottom*) Reprinted with permission from Ref. [45]. Copyright 2015 Wiley–VCH Verlag GmbH & Co

The design of microfluidic chips can enable either very fast or very slow diffusion and so dictate the speed of crystallisation of precipitation reactions in a way that cannot be achieved in batch reactors. Cao and co-workers developed a microreactor in which one channel held a well of reagent at either end (Fig. 22) [45]. By initially filling the connecting channel with oil the diffusion of the two reagents was retarded such that combination of the reagent streams, and thus crystallisation, took four days. By using the microfluidic diffusion chip to produce single crystals the first crystal structure of silver phenylacetylide was determined; silver phenylacetylide precipitates rapidly and hitherto not been isolated in a large enough single crystal for X-ray analysis.

At the opposite end of the scale, microfluidics has been widely used to achieve very fast reaction kinetics. This is most evident in flow chemistry applications in which the fast mixing of microfluidics has been used to intensify reaction processes and regulate temperature, allowing reactions to be performed in a fraction of the time [46] and at much higher temperatures [1] than is possible in traditional batch reactions. In flow crystallisation processes, fast mixing has been used largely to achieve homogeneous materials especially for anti-solvent/drowning out crystallisation conditions [1, 5, 6, 47–50]. For example the precipitation reaction of CaCO_3_ from CaCl_2_ and Na_2_CO_3_ can have three different polymorphic products which are largely dependent on initial mixing of the reagents [51]. By tuning the initial mixing conditions de Mello and co-workers were able to access selectively either the calcite or vaterite forms of CaCO_3_ using a liquid segmented microfluidic chip [52].

## Growth and nucleation studies

Because the mixing conditions can be tuned in flow crystallisation it is therefore a useful technique for evaluating growth conditions of analytes. Using a Couette-Taylor (CT) mesoreactor, Kim et al. investigated the CaCO_3_ crystal habit resulting from varying reagent ratios [53, 54]. By combining a solution of Ca(OH)_2_ with CO_2_ gas in the vortex type mixing environment of the CT reactor with varying gas: liquid ratios the habit of CaCO_3_ could be tuned. It was postulated that excess species would block the faces of growing crystals and so either spheres or cubes could be obtained by optimising the mixing conditions.

**Fig. 23:**
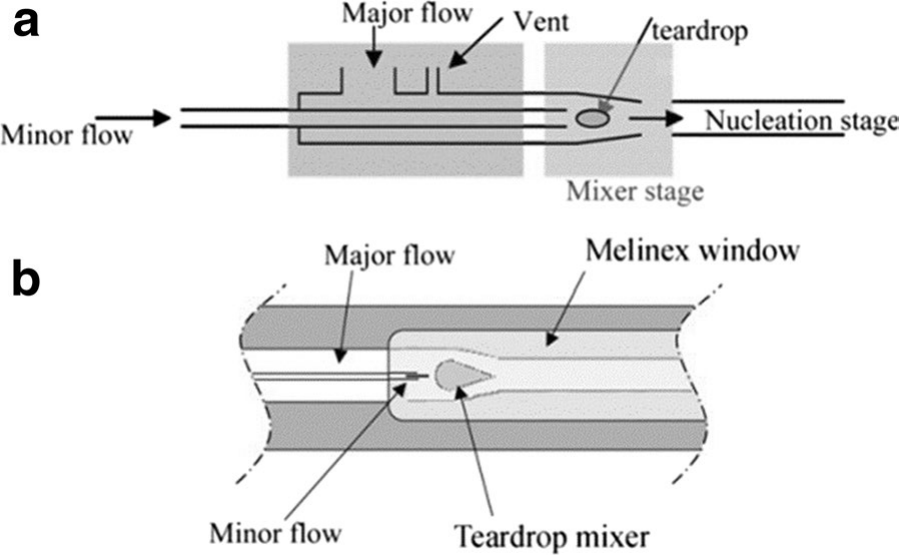
Schematic illustration of flow crystallisation set-up used for SAXS/WAXD experiments showing; **a** overview of crystalliser, **b** expanded view of impinging jet and static mixer. Reprinted with permission from Ref. [50]. Copyright 2003 The Owner Societies

The nucleation of crystallising species has been a topic of much study for many years with various nucleation mechanisms proposed [55]. Using microfluidics as a tool for producing a continual stream of homogeneous assembling particles of 2,6-dibromo-4-nitroaniline (DBA), Davey et al. investigated the nucleation of DBA with in situ SAXS/WAXD (small angle X-ray diffraction/wide angle X-ray diffraction) measurements [50]. Anti-solvent crystallisation of DBA was realised with an impinging jet type crystalliser with a static mixing obstruction (teardrop mixer) for intensified mixing (Fig. 23). Immediately downstream of this suspension SAXS/WAXD data were recorded at different distances form the mixer (0.5–10.5 cm) with a flow cell. The findings show that a SAXS signal is evident significantly upstream from the WAXD signal, implying that a non-crystalline phase is first generated before a crystalline phase emerges.

## Control of PSD and scale-up—continuous crystallisation

Whilst previous examples in this review have shown that microfluidics can deliver a narrow PSD with relative ease, this section will focus on the application of self-assembly control in reactors/crystallisers designed for scale-up applications. In order to accommodate the large-scale production of particles that are often greater than 100 μm in dimension, the design of these crystallisers have increased internal dimensions (mm–cm) with respect to those employed in microfluidics. This increase in channel size results in a corresponding decrease in mixing intensity and so alternative apparatus designs and nucleation control techniques are required to recover control of assembly conditions.

The induction of primary nucleation is driven in one of two ways: homogeneous nucleation—where solute species come together in solution to form a nucleus; or heterogeneous nucleation—where solute species adsorb onto (often microscopic) solid surfaces [56]. The former is typically concentration and mass transport driven; increasing the likelihood of collisions (through increasing density and/or velocity of solute species) increases the likelihood of sufficient species coming together to surmount the energy barrier to form a nucleus. The latter can occur due to suspended solids, e.g. impurities or already present crystals (seeds), or interaction of the solute with the crystalliser/reactor walls. The interaction with, and growth upon, crystalliser walls is termed fouling and is a significant challenge for continuous crystallisation as it threatens the homogeneity of product [57, 58]. Discussion of fouling is outside the focus of this review but it highlights the need for continuous crystallisation platforms to control the nucleation conditions in order to minimise this risk.

Control of nucleation is most easily achieved by ensuring its induction at a desired point. Nucleation induction by anti-solvent addition has been introduced in previous examples in this review, either in the form of a pure solvent in which the crystallising species is not soluble [14, 50] or using solvents in which the starting materials are soluble but reaction product is not; precipitation reactions [15, 19]. Myerson and co-workers followed the anti-solvent crystallisation of ketoconazole in a mesoreactor (3.2 mm ID) with static mixing elements [5]. By changing the flow rate the effect of mixing intensity on the nucleation and growth of ketoconazole was investigated, showing that at low flow rates (and therefore low mixing intensity) the resultant crystal size (analysed by on-line focussed beam reflection measurement—FBRM) and yield was smaller than for higher flow rates. This is contrary to expectations for standard crystallisation experiments, in which faster mixing is expected to lead to a higher number of nuclei and thus smaller crystals; in this example once nuclei are formed the crystallisation process becomes growth driven and so is dependent on mass transfer for increased crystal growth. Critically for the success of this process, the mass transfer in flow environments is more effective than in batch, thus favouring this outcome. These findings were confirmed by off-line concentration analysis.

Nucleation can be induced through acoustic cavitation using an ultrasonic device [59], in which localised regions of low pressure and high concentration result in the formation of nuclei. Using a mesoreactor with a sonic probe and subsequent air-segmentation, Myerson et al. obtained a high yield of l-aspargine monohydrate (LAM) with a narrow PSD [60]. The nucleation of LAM was controlled by the power amplitude of the sonic probe and crystal growth thereafter was controlled by cooling, smaller and more homogeneous crystals were obtained at higher power amplitudes as expected. Khinast and co-workers previously used a similar set-up but with a sonic bath rather than a sonic probe, which led to more inhomogeneity due to the increased residence time of the solution in the nucleation-inducing sonic portion of the reactor [61].

Mixing of solution feeds which are saturated at different temperatures can induce nucleation in a similar way to anti-solvent addition. The sudden drop in temperature for the hot solution results in precipitation whilst the remaining solute provides a plentiful supply of growth solution for cooling crystallisation. By combining streams of aqueous LAM solution saturated at 65 and 22 °C, Braatz and co-workers produced crystals with a narrow PSD [62]. The achievement of narrow PSD was aided by a fines dissolution mechanism of hot/cold cycling along the reactor length; the heated sections are sufficiently long to re-dissolve small crystals but not the larger ones [63, 64].

**Fig. 24:**
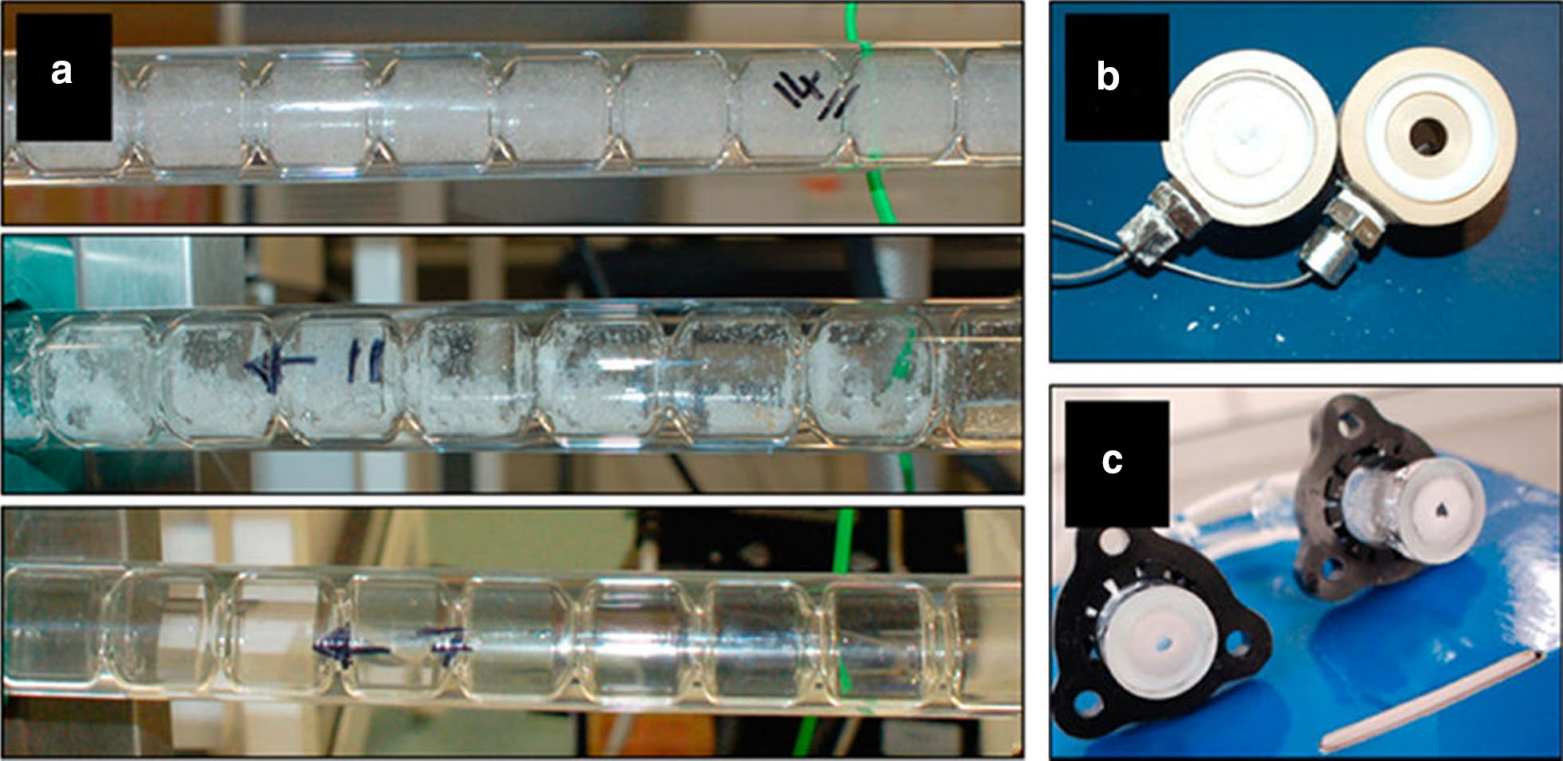
**a** Progressive encrustation during an unseeded run (*bottom* to *top*), **b** PEEK collar from COBC blocked during unseeded run compared to a clean collar (*right*), **c** encrustation in bend joints creating constriction. Reprinted with permission from Ref. [6]. Copyright 2015 American Chemical Society

The most common form of nucleation control in industry is by seeding, in which a slurry of very small crystals are pre-prepared and added to the net stream of growth solution [58, 65–69]. Using a large-scale crystalliser capable of producing kg/h product (a COBC of 15 mm ID), Florence et al. compared the crystallisation of l-glutamic acid (LGA) with and without seeding [66]. In both cases the growth of LGA was controlled by an extensive temperature regime using 13 independently controlled temperature zones. Seeds of the stable β-form of LGA were produced using a reverse jet anti-solvent crystallisation process and introduced to the crystalliser immediately downstream of the growth solution inlet. Seeded crystallisation yielded a narrow PSD of β-LGA without any fouling being observed during a 10 h run; unseeded experiments conducted as controls suffered from extensive fouling, a wide PSD and yielded exclusively the metastable α-form of LGA (Fig. 24). This polymorphic discrepancy can be explained by the different nucleation mechanism/kinetics; the seeds were produced rapidly by anti-solvent crystallisation, whereas crystals in the unseeded run were produced either slowly in solution or by secondary nucleation upon contact with the walls.

## Conclusions

Flow technologies have enabled control over self-assembled systems to be achieved in a way that is unobtainable under batch conditions. By employing very small amounts of material and/or excellent mixing conditions the concentration/ratio of reagents can be precisely controlled without concern over micromixed regions. This can be used to generate reproducible, homogeneous product or to investigate a wide range of synthesis or assembly parameters. The ordering of reagents in a flow assembly set-up is such that multi-step assembly is facile and does not require the long equilibration time required in batch. In particular, flow processing of nanoparticles is becoming very common as particle size homogeneity is of the utmost importance for these functional materials.

With the rapid development of flow technologies and their increasingly accessible cost, the use of these platforms is expanding over a wide range of chemistries and crystallisations. As more and more research groups are investigating flow methods, the pool of expertise and variety of applications available is broadening, enabling a new generation of innovative chemistry to be developed and applied.
